# Enzymatic liver function measured by LiMAx – a reliable diagnostic and prognostic tool in chronic liver disease

**DOI:** 10.1038/s41598-019-49746-1

**Published:** 2019-09-19

**Authors:** Matthias Buechter, Sarah Kersting, Guido Gerken, Alisan Kahraman

**Affiliations:** Department of Gastroenterology and Hepatology, University Clinic of Essen, University of Duisburg-Essen, Essen, Germany

**Keywords:** Ascites, Liver fibrosis, Liver cirrhosis

## Abstract

Chronic liver disease (CLD) is a major cause of morbidity and mortality worldwide. Non-invasive assessment of hepatic disease severity represents a relevant issue to further improve clinical management and therapeutic treatment. We retrospectively compared the diagnostic and prognostic performance of different non-invasive tools (LiMAx, transient elastography (TE), and biomarkers) in detecting different severity stages during the course of CLD. Patients were divided into four groups based on clinical parameters: (1) patients without CLD (control group), (2) patients suffering from CLD without having cirrhosis, (3) patients with CLD and compensated cirrhosis, and finally, (4) patients with CLD and decompensated cirrhosis. Patients with acute liver failure were excluded from the analysis. A total of 464 patients who underwent LiMAx measurement at the University Clinic of Essen between 10/2016 and 11/2017 were included in this study. Distribution of the different groups were n = 72 patients for group 1, n = 134 patients for group 2, n = 160 patients for group 3, and n = 98 patients for group 4, respectively. Median LiMAx values significantly declined with respect to increasing degree of CLD: (1) 510 µg/h/kg, (2) 390 µg/h/kg, (3) 264 µg/h/kg, and (4) 151 µg/h/kg (p < 0.001). When comparing the diagnostic accuracy of the LiMAx test in detecting patients with presence of cirrhosis (groups 1 and 2 vs. groups 3 and 4), an AUROC of 0.942 was found (cut-off 322 µg/h/kg, sensitivity 86.1%, specificity 91.3%, p < 0.0001). LiMAx was superior to TE and serum biomarkers in predicting patients’ outcome by 90-day mortality (AUROC 0.811, p < 0.001). Enzymatic liver function measured by LiMAx was closely associated with different severity stages of CLD and was a reliable diagnostic and prognostic tool with an accuracy comparable to current standard methods.

## Introduction

Chronic liver disease (CLD) is a major cause of morbidity and mortality worldwide. Due to chronic inflammation and resulting fibrosis of the parenchyma, liver cirrhosis is the clinical endpoint of CLD irrespective of its underlying etiology^1^. Resulting portal hypertension (PH), the hemodynamic abnormality defined by an increase of hepatic venous pressure gradient (HVPG) ≥6 mmHg, is the most prominent contributing factor for morbidity and mortality in this patient collective. If HVPG exceeds the threshold of 10 mmHg, designated as “clinical significant portal hypertension” (CSPH), it is likely that the most serious complications of cirrhosis, including ascites, hepatic encephalopathy, and gastroesophageal variceal bleeding arise (“decompensated portal hypertension (DPH)”)^2,3^. Despite diagnostic and therapeutic improvements during the last decades, one-year mortality is still in the order of 20–60% among patients with DPH^4^.

Assessment of hepatic disease severity represents a relevant issue to further improve clinical management and therapeutic treatment. Several serum biomarkers, liver indices, and scoring systems have been proposed to overcome this problem and determine prognosis. The most established and widely used are Child-Pugh Classification (CPS) and Model of End-Stage Liver Disease (MELD) score^5,6^. Furthermore, mathematical scoring systems combining serum biomarkers such as aspartate aminotransferase (AST) to alanine aminotransferase (ALT) ratio (AAR), AST-to-platelet ratio index (APRI), and fibrosis-4 (FIB-4) score have been developed to stage CLD and determine disease severity^7–9^. In addition, liver stiffness measurement by transient elastography (TE; Fibroscan) - a technology based on measuring the velocity of a shear wave by a mechanical impulse - has gained attention over the last years^10,11^.

Recently, measurement of the enzymatic liver function by liver maximum capacity (LiMAx) breath test has been introduced as a solid technique to determine dynamic liver function based on the specific hepatic cytochrome P450 1A2 metabolism of an intravenously injected substance (^13^C-methacetin). The LiMAx test was successfully evaluated in several clinical settings such as liver surgery, acute liver failure, liver transplantation, sepsis, and liver cirrhosis^12–20^. In our study, we aimed to assess the diagnostic and prognostic accuracy of different non-invasive tools ([1] LiMAx, [2] TE, [3] CPS, [4] MELD, [5] AAR, [6] APRI, [7] FIB-4, and [8] spleen size) to stage CLD in a large cohort of 464 patients.

## Patients and Methods

### Study population

The study population consisted of patients with CLD of various etiologies who were treated at the Department of Gastroenterology and Hepatology (University Clinic of Essen, Germany) between October 2016 and December 2017. Patients with acute liver failure were excluded from the analysis. Based on clinical, laboratory, ultrasound, endoscopic, and - if available - histological criteria (n = 184/464, 39.6%), patients were classified into four groups:

- group I: patients without (history of) CLD (control group)
- group II: patients with CLD without cirrhosis
- group III: patients with CLD and compensated cirrhosis (cirrhosis with absence of decompensation signs)
- group IV: patients with CLD and decompensated cirrhosis (at least one of the following)

- presence of hepatic ascites ± spontaneous bacterial peritonitis
- presence of portal hypertension (PH)-induced gastrointestinal/variceal hemorrhage
- presence of renal dysfunction/hepatorenal syndrome (HRS)
- presence of hepatic encephalopathy (HE)

The study was conducted in accordance with the Declaration of Helsinki. The protocol was approved by the ethics committee of the University of Duisburg-Essen.

### Non-invasive tools for the assessment of chronic liver disease

Inquiry of data for non-invasive assessment of CLD as stated below was performed within 24 hours of the LiMAx measurement.

#### Liver maximum capacity (LiMAx)

LiMAx test (Humedics, Berlin, Germany) was performed after a minimum of 3 h fasting. The measurement is based on hepatocellular-specific metabolism of intravenously administered ^13^C-labeled methacetin - an exclusive substrate for the hepatic cytochrome P450 1A2 enzyme. ^13^C-methacetin in hepatocytes is immediately demethylated into acetaminophen and ^13^CO_2_; the latter is subsequently exhaled, leading to an increase of ^13^CO_2_ concentration in breath. Prior to substrate injection, the individual baseline ratio ^13^CO_2_/^12^CO_2_ concentration of a patient is measured and thus liver function capacity can be calculated from the analysis of the ^13^CO_2_/^12^CO_2_ ratio within 60 minutes after injection. Results are given in µg/h/kg.

#### Serum biomarkers and CLD scoring systems

The following laboratory investigations for calculating different indexes and scoring systems were evaluated: aspartate aminotransferase (AST) to alanine aminotransferase (ALT) ratio (AAR), AST-to-platelet ratio index (APRI), fibrosis-4 (FIB-4) score, Model of End Stage Liver Disease (MELD) Score, and Child-Pugh Score (CPS). These were calculated as listed below:

- AAR: AST/ALT [U/L]
- APRI: (AST [U/L]/upper limit of normal range [U/L])/(platelet count [10^9^/L] x 100)
- FIB-4: (age [years] x AST [U/L])/ (platelet count [10^9^/L] x ALT [U/L])
- MELD score: 10 * (0,957 * ln(creatinine) + 0,378 * ln(bilirubin) + 1,12 * ln(INR) + 0,643)
- CPS: total serum bilirubin level [mg/dL] (<2.0: 1 point; 2.0–3.0: 2 points; >2.0: 3 points) + serum albumin [g/dL] (>3.5: 1 point; 2.8–3.5: 2 points; <2.8: 3 points) + INR (<1.7: 1 point; 1.7–2.2: 2 points; >2.2: 3 points) + ascites (none: 1 point; mild: 2 points; moderate to severe: 3 points) + HE (none: 1 point; grade I-II: 2 points; grade III-IV: 3 points according to West Haven criteria^21,22^).

#### Liver stiffness measurement (LSM) by transient elastography (TE) and spleen size measurement (SSM)

LSM (Fibroscan, Echosens, Paris, France) was performed after fasting for at least 8 h pre-exam and with the patient in supine position with arms in maximal abduction. The standard TE probe (type M) was used via intercostal spaces on the right lobe of the liver. In obese patients, the XL-probe was applied. Each examination was assisted by B-mode ultrasound (APLIO 500, Toshiba, Japan) for identification of feasible probe position and exclusion of perihepatic ascites. The median of at least 10 LSM values expressed in kilopascal (kPa) was used as the representative measurement. Success rate was calculated as number of valid measurements divided by number of total measurements. According to the manufacturer’s recommendation and published evidence, only patients with an interquartile range (IQR) < 30% of the median value and a success rate >60% were included in the analysis^23^. LSM and SSM were performed by an experienced observer who had performed at least 500 examinations each. Ninety-nine patients (21.3%) had to be excluded due to technical reasons (e.g., ascites, IQR > 30%, success rate <60%). Measurement of the spleen size was accomplished by ultrasound along its long axis and expressed in cm. In 42/464 patients (0.9%), spleen size was not available for analysis.

### Statistical analysis

All analyses were done by a professional statistician using the statistical analysis software SAS 9.4. Data from 464 subjects were used. Median values are reported with interquartile range (IQR) while mean values are reported with standard deviation and range (i.e. Min/Max). Area under receiver operating characteristics (AUROCs) are given along with 95% confidence interval (CI). The vbox-statement of PROC SGPLOT was utilized for boxplots in which the rhomb represents the mean value, the vertical line the median, the box the IQR, and the whiskers the last observation within a range of 1.5 x IQR. Dots in the plots mark outliers. The Wald confidence interval was used for the 95% confidence interval of AUROC.

For receiver operating characteristics (ROC) curves, the ROC-statement of PROC LOGISTICS was used. The model specified for the ROC curve included the classification system for the clinical severity stage as dependent variable and the liver test of interest as independent variable. 95% confidence intervals were obtained by the ROCCONTRAST statement which uses the Wald method by default for calculation. In general, optimal cut-offs were determined by the highest Youden index which corresponds to the sum of sensitivity and specificity minus 1.

### Ethics approval and consent to participate

The ethics committee of the University of Duisburg-Essen approved the anonymous collection and publication of the data.

## Results

### Patients’ characteristics

A total of 464 subjects were included in this study. Mean age of the study population was 58.5 ± 14.2 (18.2–85.6) years, while the majority was male (n = 279; 60.1%). According to the above described classification, 72 patients (15.5%) were assigned to group I (control group), 134 (28.9%) to group II, 160 (34.5%) to group III, and 98 (21.1%) to group IV, respectively. The etiologies leading to CLD (groups II-IV, n = 392) were in detail: alcohol-induced liver disease (n = 91; 23.2%), non-alcoholic steatohepatitis (NASH; n = 91; 23.2%), autoimmune hepatitis (AIH; n = 47; 12.0%), chronic hepatitis C virus infection (HCV; n = 41; 10.5%), chronic hepatitis B virus infection (HBV; n = 33; 8.4%), primary sclerosing cholangitis (PSC; n = 26; 6.6%), primary biliary cholangitis (PBC; n = 20; 5.1%), cryptogenic liver disease (n = 20; 5.1%), chemotherapy induced liver disease (n = 9; 2.3%), hemochromatosis (n = 6; 1.5%), hepatic sarcoidosis (n = 3; 0.8%), secondary sclerosing cholangitis (SSC; n = 3; 0.8%), and Wilson’s disease (n = 2; 0.5%).

### Correlation between diagnostic tests and CLD stages

Median values of the different diagnostic tests according to CLD stages are demonstrated in Table 1. The Spearman’s rank correlation test showed a statistically significant association between all diagnostic tests and the different stages of CLD (Spearman’s r for [1] LiMAx: −0.81, [2] TE: 0.75, [3] CPS: 0.64, [4] MELD score: 0.61, [5] FIB-4: 0.60, [6] AAR: 0.56, [7] APRI: 0.48, and [8] spleen size: 0.45, p < 0.0001 for all correlation coefficients).

**Table 1:** Median values of non-invasive tests according to clinical stages among patients with chronic liver disease (n = 464).

Diagnostic test	Group I	Group II	Group III	Group IV	Spearman’s r
LiMAx, µg/h/kg	509.5 (410.5; 570.0)	390.0 (343.0; 453.0)	264.0 (215.0; 308.0)	150.5 (96.0; 219.0)	−0.81
TE, kPa	5.6 (4.0; 9.1)	7.6 (4.9; 10.2)	27.0 (17.3; 41.0)	54.2 (29.9; 75.0)	0.75
CPS	5 (5; 5)	5 (5; 5)	5 (5; 7)	8 (7; 9)	0.64
MELD score	7 (6; 9)	7 (6; 8)	10 (8; 12)	15 (10; 20)	0.61
FIB-4 score	1.54 (0.97; 2.23)	1.15 (0.79; 1.95)	4.63 (2.92; 8.36)	5.53 (2.89; 10.58)	0.60
AAR	0.90 (0.75; 1.30)	0.80 (0.60; 1.10)	1.30 (1.00; 1.85)	1.90 (1.50; 2.40)	0.56
APRI	0.30 (0.20; 0.45)	0.40 (0.20; 0.70)	1.00 (0.60; 1.80)	1.15 (0.50; 2.60)	0.48
spleen size, cm	10.4 (9.2; 11.9)	10.4 (9.0; 12.2)	13.5 (11.5; 16.5)	13.5 (11.4; 15.3)	0.45

Data represents median and IQR.

*AAR, AST/ALT ratio; APRI, AST-to-platelet-ratio index; CPS, Child Pugh Score; FIB4, fibrosis-4; LiMAx, liver maximum capacity; MELD, model of end-stage liver disease; TE, transient elastography.

### Diagnostic accuracy of the different tests in detecting CLD (group I vs. group II-IV)

**Figure 1:**
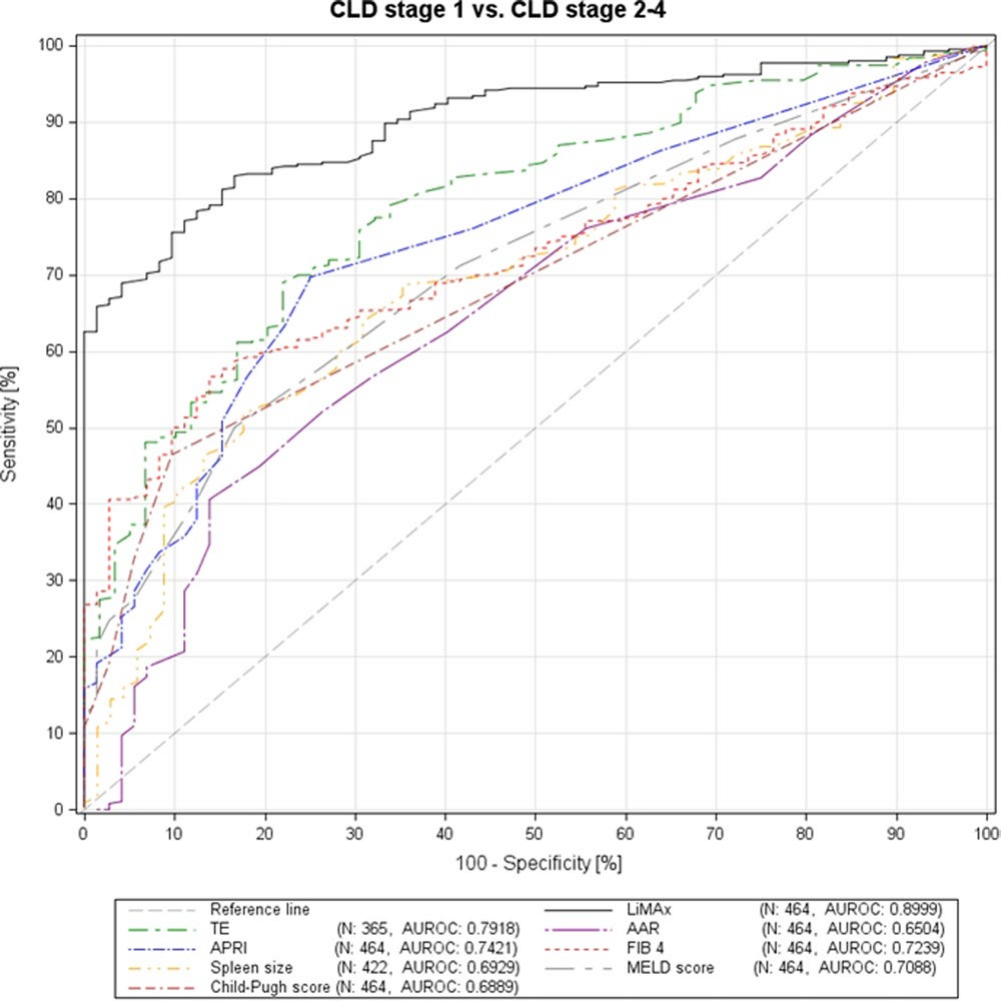
Receiver operating characteristics (ROC) curves for the different non-invasive tests in detecting patients with chronic liver disease (group I vs. group II-IV).

LiMAx had the highest diagnostic accuracy in detecting patients with CLD (AUROC 0.8999, sensitivity 82.91%, specificity 83.33%, PPV 96.44%, NPV 47.24%, cut off 398 µg/h/kg, Youden index 0.66) and was superior to TE (AUROC 0.7918, sensitivity 68.95%, specificity 77.97%, PPV 94.20%, NPV 32.62%, cut off 9.3 kPa, Youden index 0.47); APRI (AUROC 0.7421, sensitivity 69.64%, specificity 75.00%, NPV 93.81%, NPV 31.21%, cut off 0.45, Youden index 0.45); FIB-4 (AUROC 0.7239, sensitivity 56.63%, specificity 86.11%, PPV 95.69%, NPV 26.72%, cut off 2.75, Youden index 0.43); CPS (AUROC 0.6889, sensitivity 46.43%, specificity 90.28%, PPV 96.30%, NPV 23.64%, cut off 6, Youden index 0.37); spleen size (AUROC 0.6929, sensitivity 51.69%, specificity 82.35%, PPV 93.85%, NPV 24.67%, cut off 12.3 cm, Youden index 0.34); MELD (AUROC 0.7088, sensitivity 50.00%, specificity 83.33%, PPV 94.23%, NPV 23.44%, cut off 10, Youden index 0.33); and AAR (AUROC 0.6504, sensitivity 40.56%, specificity 86.11%, PPV 94.08%, NPV 21.02%, cut off 1.5, Youden index 0.27), respectively. ROC curves of the different diagnostic tests for detecting patients with CLD are demonstrated in Fig. 1.

### Diagnostic accuracy of the different tests in detecting liver cirrhosis (group I-II vs. group III-IV)

**Figure 2:**
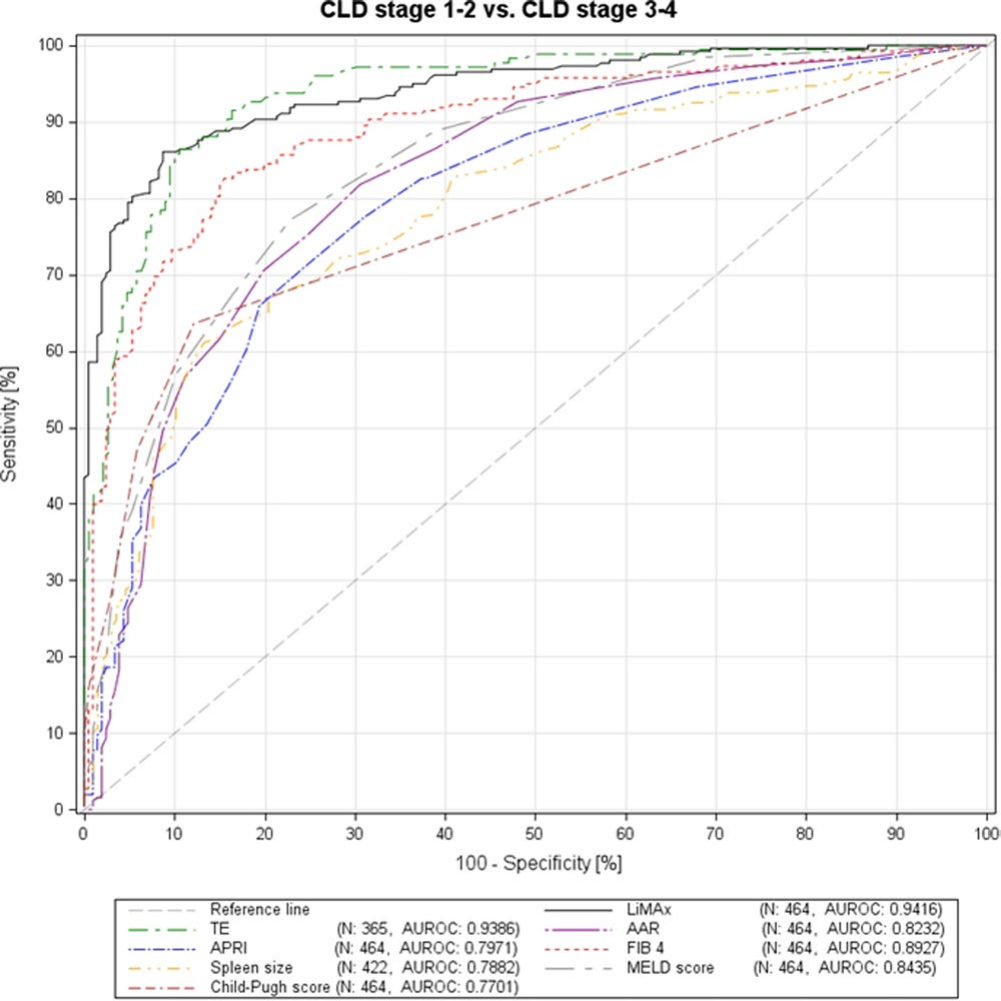
ROC curves for the different non-invasive tests in detecting patients with liver cirrhosis (group I-II vs. group III-IV).

For the detection of liver cirrhosis, the diagnostic accuracy of LiMAx (AUROC 0.941, sensitivity 86.05%, specificity 91.26%, PPV 92.50%, NPV 83.93%, cut off 322 µg/h/kg, Youden index 0.77) and TE (AUROC 0.9386, sensitivity 86.36%, specificity 89.42%, PPV 88.37%, NPV 87.56%, cut off 15 kPa, Youden index 0.76) were equivalent and superior compared to FIB-4 (AUROC 0.8927, sensitivity 82.56%, specificity 84.47%, PPV 86.94%, NPV 79.45%, cut off 2.63, Youden index 0.67); MELD (AUROC 0.8435, sensitivity 77.13%, specificity 77.18%, PPV 80.89%, NPV 72.94%, cut off 9, Youden index 0.54); CPS (AUROC 0.7701, sensitivity 63.57%, specificity 87.86%, PPV 86.77%, NPV 65.82%, cut off 6, Youden index 0.51); AAR (AUROC 0.8232, sensitivity 81.78%, specificity 69.42%, PPV 77.01%, NPV 75.26%, cut off 1.1, Youden index 0.51); spleen size (AUROC 0.7882, sensitivity 61.06%, specificity 86.73%, PPV 84.15%, NPV 65.89%, cut off 13.0 cm, Youden index 0.48); and APRI (AUROC 0.7971, sensitivity 71.32%, specificity 75.24%, PPV 78.30%, NPV 67.69%, cut off 0.7, Youden index 0.47) (Fig. 2).

### Diagnostic accuracy of the different tests in detecting decompensated liver cirrhosis (group I-III vs. group IV)

**Figure 3:**
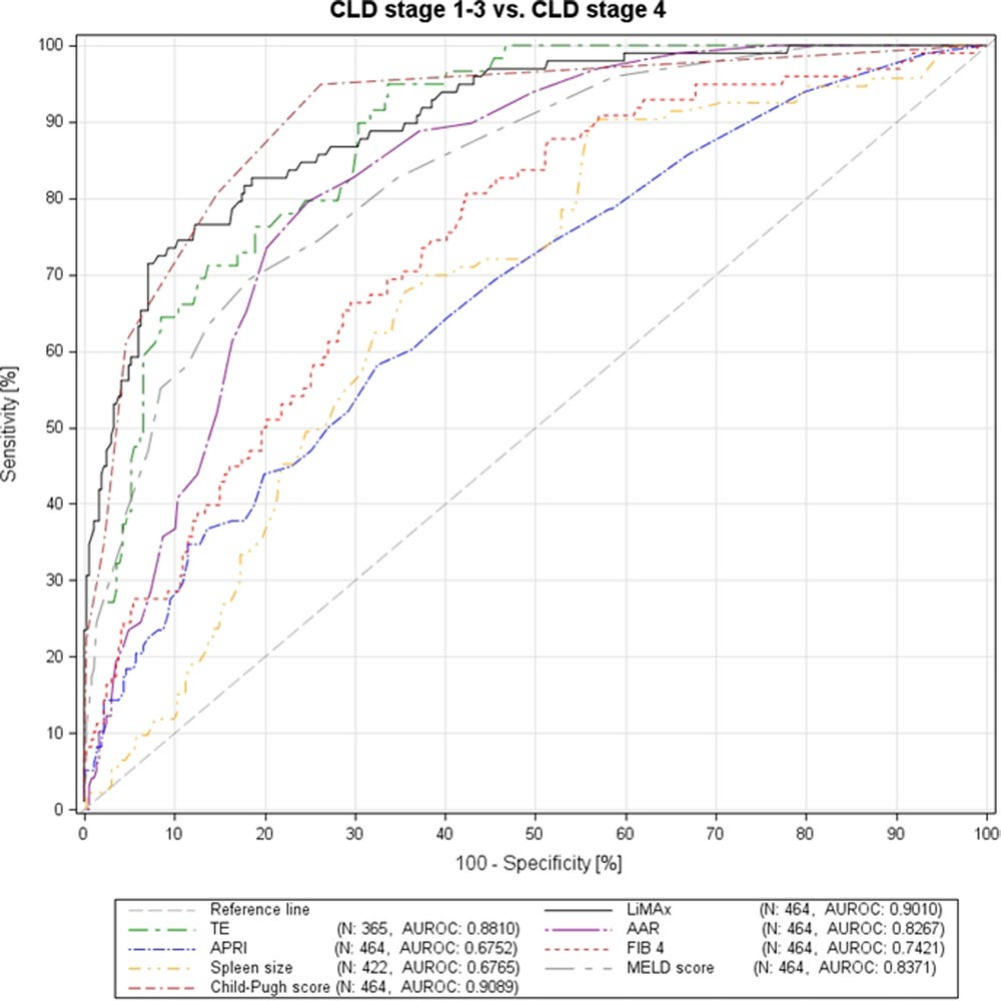
ROC curves for the different non-invasive tests in detecting patients with decompensated liver cirrhosis (group I-III vs. group IV).

The diagnostic performance of the different tests in detecting decompensated cirrhosis did not significantly differ for CPS (AUROC 0.9089, sensitivity 94.90%, specificity 73.77%, PPV 49.21%, NPV 98.18%, cut off 6, Youden index 0.69); LiMAx (AUROC 0.9010, sensitivity 71.43%, specificity 92.90%, PPV 72.92%, NPV 92.39%, cut off 194 µg/h/kg, Youden index 0.64); and TE (AUROC 0.8810, sensitivity 94.92%, specificity 66.34%, PPV 35.22%, NPV 98.54%, cut off 17.7 kPa, Youden index 0.61). CPS, LiMAx and TE were superior in detecting patients with decompensated cirrhosis than AAR (AUROC 0.8267, sensitivity 79.59%, specificity 75.14%, PPV 46.15%, NPV 93.22%, cut off 1.5, Youden index 0.55); MELD (AUROC 0.8371, sensitivity 69.39%, specificity 81.69%, PPV 50.37%, NPV 90.88%, cut-off 12, Youden index 0.51); FIB-4 (AUROC 0.7421, sensitivity 80.61%, specificity 57.65%, PPV 33.76%, NPV 91.74%, cut off 2.72, Youden index 0.38); spleen size (AUROC 0.6765, sensitivity 89.25%, specificity 44.07%, PPV 31.09%, NPV 93.55%, cut off 10.9 cm, Youden index 0.33); and APRI (AUROC 0.6752, sensitivity 58.16%, specificity 67.49%, PPV 32.39%, NPV 85.76%, cut off 1, Youden index 0.26), respectively. ROC curves for the different testes in detecting decompensated cirrhosis are illustrated in Fig. 3.

### Prognostic accuracy of the different test (90-days mortality)

**Figure 4:**
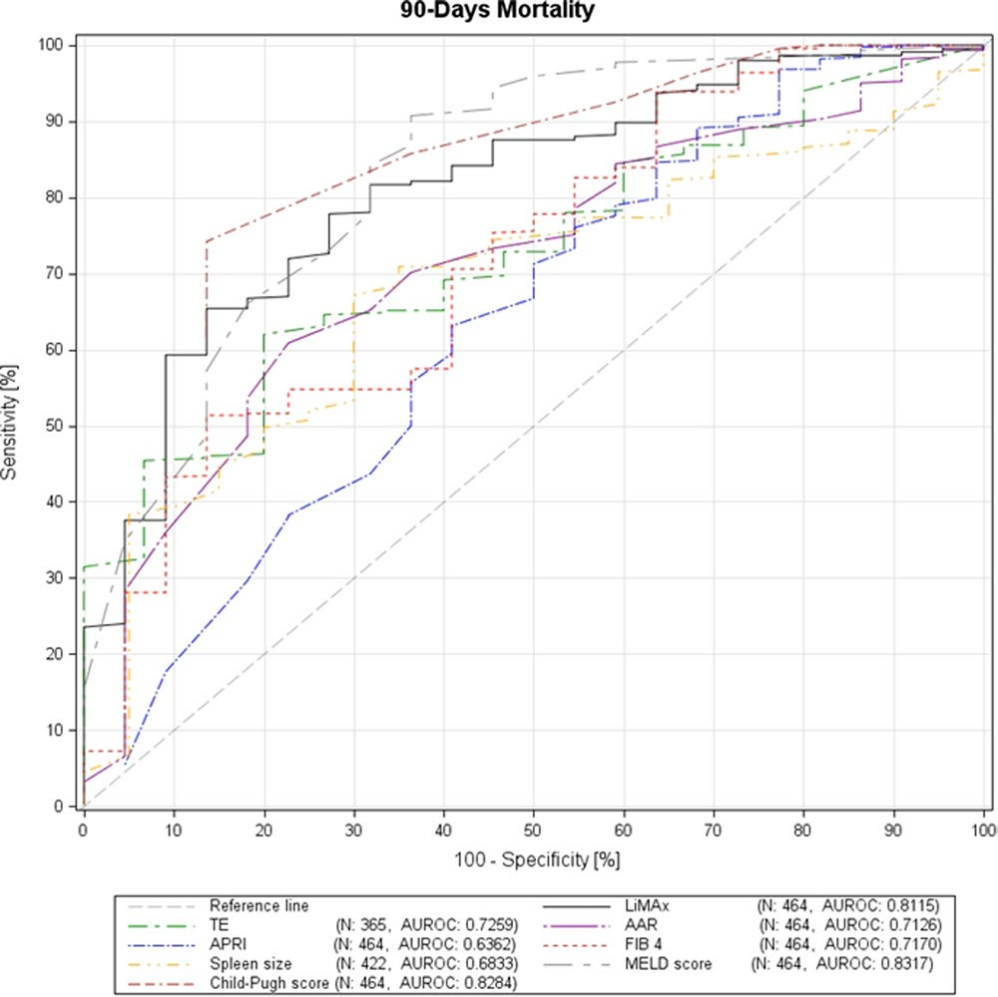
ROC curves for the different non-invasive tests predicting 90-day mortality.

Our patient collective was further stratified according to the 90-days mortality. As expected, the mortality rate exponentially increased according to progressing CLD (group I: 0/76 (0.0%), group II: 1/134 (0.7%), group III: 6/160 (3.8%), and group IV: 15/98 (15.3%)). Interestingly, the accuracy of LiMAx (AUROC 0.8115, sensitivity 65.38%, specificity 86.36%, PPV 98.97%, NPV 11.05%, cut off 268 µg/h/kg, Youden index 0.52) was comparable to CPS (AUROC 0.8284, sensitivity 74.21%, specificity 86.36%, PPV 99.09%, NPV 14.29%, cut-off 6, Youden index 0.61), and MELD (AUROC 0.8317, sensitivity 90.72%, specificity 63.34%, PPV 98.04%, NPV 25.45%, cut off 16, Youden index 0.54), which represent scoring systems exclusively developed for this purpose, in predicting 90-days mortality. The prognostic accuracy of TE (AUROC 0.7259, sensitivity 62.00%, specificity 80.00%, PPV 98.64%, NPV 8.28%, cut off 21.1 kPa, Youden index 0.42); AAR (AUROC 0.7126, sensitivity 60.86%, specificity 77.27%, PPV 98.18%, NPV 8.95%, cut off 1.3, Youden index 0.38); FIB-4 (AUROC 0.7170, sensitivity 51.36%, specificity 86.36%, PPV 98.70%, NPV 8.12%, cut off 2.7, Youden index 0.38); spleen size (AUROC 0.6833, sensitivity 67.16%, specificity 70.00%, PPV 97.83%, NPV 9.59%, cut off 16 cm, Youden index 0.37); and APRI (AUROC 0.6362, sensitivity 63.12%, specificity 59.09%, PPV 96.88%, NPV 7.39%, cut off 0.9, Youden index 0.22) was significantly worse (Fig. 4).

## Discussion

Non-invasive identification of patients with advanced stages of chronic liver disease (CLD) is clinically important as it serves as a relevant determining factor in the assessment of disease progression and long-term outcome. Today, different diagnostic tools are used for estimation of hepatic disease severity and individual prognosis. Our study focused on the measurement of hepatic function in CLD using the LiMAx test. LiMAx has been previously evaluated in various clinical settings such as liver surgery, liver transplantation, cirrhosis, oncology, acute liver failure, sepsis, and drug management^12–20,24,25^. Hence, we applied the test to determine enzymatic liver function in a large cohort of 464 patients with CLD. We hypothesized that the LiMAx test could provide additional information on disease severity, possibly allowing better stratification of these patients. We therefore compared the diagnostic and prognostic performance of the LiMAx test with established serum indices, clinical scoring systems, and transient elastography (TE).

**Figure 5:**
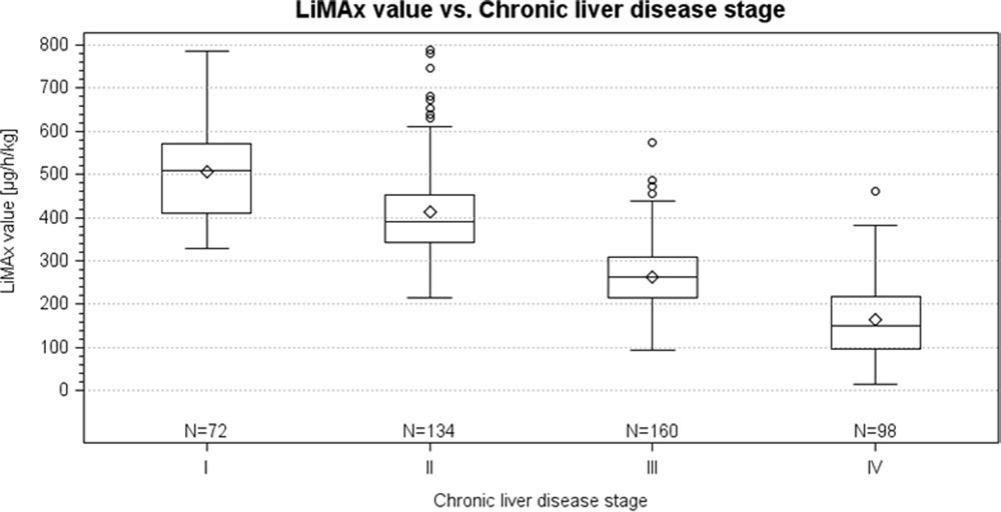
Correlation between liver maximum capacity (LiMAx) and clinical stages of CLD (n = 464).

Enzymatic liver function measured by LiMAx showed strong correlation with different clinical stages occurring in the course of CLD with a Spearman’s correlation coefficient of −0.81 and was superior to TE (Spearman’ s r 0.75), CPS (Spearman’s r 0.64), MELD (Spearman’s r 0.61), and serum biomarkers (Spearman’s r 0.45–0.56). Median LiMAx values showed clear decreasing tendencies with respect to progression of CLD (group I: 509.5 (410.5; 570.0) µg/h/kg, group II: 390.0 (343.0; 453.0) µg/h/kg, group III: 264.0 (215.0; 308.0) µg/h/kg, and group IV: 150.5 (96.0; 219.0) µg/h/kg, p < 0.0001) as demonstrated in Fig. 5. The superiority of LiMAx, however, is not essentially surprising: the applied substrate (^13^C-methacetin) is metabolized by the cytochrome P450 1A2 enzyme - which is expressed in hepatocytes exclusively. In a recently published work of our group, we could demonstrate that structural changes in the course of CLD (reflected by different fibrosis-stages) were closely associated with changes of enzymatic liver function measured by LiMAx (Spearman’s r −0.68)^13^. In contrast, routine laboratory parameters - used for the calculation of above-mentioned serum indices - are not liver-specific and released in various organs of the human body. Furthermore, these parameters are influenced by factors such as iatrogenic substitution, or biological half-life time.

Liver cirrhosis is the clinical endpoint of CLD irrespective of its underlying etiology. Development of portal hypertension (PH), defined by HVPG values ≥6 mmHg, is the key factor in the natural history of CLD and represents the main driver of complications. Ongoing disease progression exceeding the threshold of 10 mmHg (“clinical significant portal hypertension” (CSPH)) is highly associated with risk of clinical decompensation events (ascites, variceal bleeding, jaundice, and hepatic encephalopathy) tremendously increasing morbidity, and mortality^2–4^. Acquisition of histology by liver biopsy for the detection of cirrhosis and determination of the HVPG for PH assessment are considered as diagnostic gold-standards among patients with CLD^3,26–28^. However, due to their invasiveness, these methods are not applicable in the daily clinical setting. Thus, non-invasive markers for the determination of hepatic disease severity have been subject of extensive scientific research during the last decades. Nonetheless, diagnostic accuracy of AAR, APRI, and FIB-4 for detecting liver cirrhosis is poor with AUROCs ranging from 0.52–0.72 depending on CLD etiology^29–33^. Liver stiffness measurement by transient elastography (TE) is probably the most validated non-invasive technique for the detection of hepatic fibrosis and cirrhosis. According to current literature, AUROC values for detection of cirrhosis (F4) range between 0.80 and 0.98^10,34,35^. Liver stiffness, however, is affected by various (patho)physiological conditions such as hepatic inflammation, congestion, mechanical cholestasis, and food intake^34^. Furthermore, in a significant number of patients, valid stiffness measurements are not obtainable due to technical reasons and/or presence of ascites (n = 99/464; 21.3% in our patient collective). Our results are consistent with those from current literature: LiMAx^®^ and TE (both AUROCs 0.94) were superior in detecting cirrhosis when compared to serum biomarkers (AUROCs 0.77–0.89) (Fig. 2). The determined cut off values for LiMAx (322 µg/h/kg) and TE (15 kPa) were comparable to those recently published in a histologically-proven cohort of our group including 102 patients^13^.

Apart from diagnostic purposes, prognostic factors and scoring systems have been investigated and (partly) implemented in clinical guidelines. Among patients with advanced CLD and cirrhosis, PH is the main determinant of complications, increasing the mortality of this patient collective. Measurement of HVPG is the gold standard for its evaluation, and the severity of PH is directly correlating with the long-term prognosis of cirrhosis^36^. However, due its invasiveness and indispensable operators’ expertise, its use is restricted mainly to tertiary centers. CPS and MELD score are the most common and widely used non-invasive models for outcome prediction in cirrhotic patients with good prognostic accuracy^37–40^. Consequently, MELD and CPS showed reliable results in predicting 90-day mortality in our collective (AUROCs of 0.8317 for MELD and 0.8284 for CPS, respectively) and were superior to TE (AUROC 0.7259), and serum biomarkers (AUROCs 0.6362–0.7170). Malinowski *et al*. already showed strong negative correlation of LiMAx with CPS (r = −0.707, p < 0.001) and MELD score (r = −0.686, p < 0.001) in their cohort of 347 cirrhotic patients^19^. Furthermore, Jara and colleagues demonstrated that LiMAx was, apart from serum creatinine levels, an independent predictor of short-term mortality in patients with liver cirrhosis (n = 268, AUROC 0.75)^14^. Accordingly, in our patient collective, the prognostic accuracy of LiMAx for 90-day mortality was excellent with an AUROC of 0.8115 and comparable to CPS (AUROC 0.8284) and MELD (AUROC 0.8317).

Besides its powerful diagnostic and prognostic validity, LiMAx represents a safe and robust non-invasive examination: no adverse events or test failures were documented during the 464 measurements. It is even possible to perform the test in mechanical ventilated patients or patients with extracorporeal membrane oxygenation devices^16,24,41^.

However, it should be mentioned that our study has various limitations. At first, histology was not available for all patients and exact classification of groups I-IV might be slightly modified. Secondly, TE was not obtainable in all patients and the actual results for liver stiffness measurement might be slightly better. Furthermore, this was a retrospectively performed single-center cohort study. However, our present findings confirm the results of our previously published histology-proven study for LiMAx and TE in large patient cohort. A prospective multicenter study would be desirable to further validate the potential of LiMAx in the assessment of disease severity in patients with CLD.

In conclusion, the present study demonstrates that enzymatic liver function was closely associated with different clinical stages occurring during the course of CLD. Our results indicate that LiMAx is not only an accurate diagnostic, but also a valuable prognostic tool in patients with CLD comparable to current standard methods.

## Data Availability

Datasets used and/or analyzed during the current study are available from the corresponding author on reasonable request.
